# Resident operative time as an independent predictor of early post-operative cataract surgery outcomes and supervising attending surgeon impact: a retrospective case series

**DOI:** 10.1186/s12886-023-03278-5

**Published:** 2024-01-10

**Authors:** Hans W Andrews, George T Lin, Jennifer L Lindsey, Xiangyu Ji, Qingxia Chen, Amy S Chomsky

**Affiliations:** 1 Vanderbilt Eye Institute, 2311 Pierce Ave, Nashville, TN 37232 USA; 2 Vanderbilt University School of Medicine, 2209 Garland Ave, Nashville, TN 37232 USA; 3 VA Tennessee Valley Healthcare System, 1310 24th Ave S, Nashville, TN 37212 USA; 4 Department of Biostatistics, Vanderbilt University Medical Center, 2525 West End, Nashville, TN 37232 USA

**Keywords:** Resident, Surgery, Cataract, Attending, Education, Operative, Time, Phacoemulsification

## Abstract

**Background:**

The authors sought to determine if resident operative time in cataract extraction and intraocular lens insertion (CE/IOL) affects early visual outcomes and post-operative recovery. They further sought to investigate if attending surgeons can reduce resident operative time.

**Methods:**

This retrospective, chart-review, case series at single Veterans Affairs Hospital (VA Tennessee Valley Healthcare System) studied resident cataract surgeries between March 1, 2018 and March 31, 2020. Following power analysis, 420 eyes of 400 patients from all resident cataract surgeries were included. Eyes with attending as primary surgeon, laser-assisted cataract surgery, or concurrent secondary procedures were excluded. Linear mixed effect models were used to study the association between operative time and visual outcomes while adjusting for covariates including cumulative dissipated energy, preoperative factors, and intraoperative complications.

**Results:**

Longer operative time was statistically associated with worse post-operative-day 1 (POD1) pinhole visual acuity (PH-VA) adjusting for cumulative dissipated energy and other operative factors (*p* = 0.049). Although resident physicians were the primary surgeons, the operative times were different between the ten supervising attending surgeons in the study (*p* < 0.001).

**Conclusion:**

The results suggest that increased resident operative time is a significant, independent risk factor for decreased POD1 PH-VA. Increased resident operative time is not associated with worsened long term visual outcomes. Attending surgeons may be able to reduce resident operative time, which is associated with improved early visual outcomes.

## Background

Cataract surgery is one of the most common surgeries performed by ophthalmologists in the United States. For most, the goal of cataract surgery is to enhance patient quality of life through improving visual acuity. With the advent of phacoemulsification, excellent early post operative outcomes have become expected by both physicians and patients. Given these high expectations for visual outcomes, it is important for trainee cataract surgery to facilitate strong visual outcomes.

Studies have suggested resident participation in cataract surgery does not affect long term outcomes, however this is debated in the literature [1–3]. Cataract surgery performed by a trainee is significantly longer than that performed by an attending physician, especially in the early months of training [4]. This can lead to intraoperative patient discomfort, delayed care for patients, and increase cost for the health care system [5]. With limited resources, like that at the United States Veterans Affairs, this means veterans may undergo long wait times or be sent out to the community for expedited care, therefore increasing the tax burden on citizens. The relationship between long operative time and visual outcomes, however, remains poorly understood.

It is well understood that increased cumulative dissipated energy (CDE) and phacoemulsification time is associated with post operative endothelial cell loss and therefore early post operative corneal edema [6, 7]. This can result in decreased post operative visual acuity (VA) at post operative day one (POD1), and post operative week one (POW1) visits [8]. However, little has been published on the effect of the total operative length *independent* from CDE or phacoemulsification time. The authors hypothesize that increased operative time is associated with decreased POD1 visual acuity and increased corneal edema, while controlling for other operative factors including CDE, pupil dilation, and surgeon experience, among others.

As a secondary outcome the authors further hypothesize that the attending surgeon may impact operative time. Studies have shown complication rates may differ between junior and senior surgeons [9]. However, the attendings effect on resident operative time has not been addressed to the best of the authors knowledge, nor have any studies assessing resident operative outcomes controlled for attending differences.

## Methods

### Study design

A retrospective review was performed on eyes that underwent level 2 cataract surgery (attending scrubbed and resident performing surgery) at a single Veterans Affairs Hospital between March 1, 2018 and March 31, 2020. The study was reviewed and approved by the Tennessee Valley Healthcare System (TVHS) Veterans Affairs (VA) Institutional Review Board and adhered to the tenets of the Declaration of Helsinki. A waiver of documentation of informed consent was granted by the TVHS VA Institutional Review Board for this study.

An initial chart review of 100 cases was performed followed by a power analysis. It was determined to reach a power of 0.8 for POD1 visual acuity outcomes, 327 charts were needed. A chart review was thusly performed on 420 eyes of 400 patients. Eyes were included if the cataract surgery was performed by a resident. Eyes that underwent secondary procedures including tube shunts, minimally invasive glaucoma surgery, pterygium removal, etc. or surgeries that were completed by attending physicians were excluded. Basic descriptive data were collected including age, laterality, dilation, pre-op visual acuity, nuclear sclerosis grade, and concomitant vision-limiting diagnoses. Documented vision-limiting diagnoses included age related macular degeneration, optic neuropathy, corneal scarring, retinal vein or arterial occlusions, Fuchs’ dystrophy, diabetic macular edema, epiretinal membrane, and amblyopia among others. Operative events were recorded including the use of epinephrine, use of trypan blue, use of iris expansion device, wound sutures, CDE, intraoperative floppy iris syndrome (IFIS), operative time, and intra-operative complication. Operative time was defined as the time between first incision and closure of the wounds.

Primary outcome variables included pinhole POD1 and best corrected POW1 VA, POD1 and POW1 corneal edema, and POD1 reported eye pain or discomfort. Corneal edema was defined by the operating physician at POD1 and POW1 as trace (1), mild (2), moderate (3), or severe (4). POD1 and POW1 anterior chamber inflammation was recorded using the 1–4 + defined by Standardization of Uveitis Nomenclature (SUN) classification, with 0.5 + used for “trace” cell. Snellen visual acuity was converted to logMAR, using 2 for count fingers acuity, 3 for hand motion, and 4 for light perception [10]. For nuclear sclerotic grading and anterior chamber inflammation, if the physician recorded 1–2+, this was recorded as 1.5. Gradings of 2–3 + were recorded as 2.5 and gradings of 3–4 + as 3.5.

Lastly the cases were reviewed for which attending was involved in the case. The attendings were de-identified and numbered and operative times were compared amongst attending surgeons. The cases were controlled for above pre-operative higher risk characteristics and operative factors.

### Statistics

Continuous variables were summarized in mean, median, standard deviation, and interquartile ranges. Categorical variables were summarized in count and frequency. Linear mixed effect models were used to study the association between operative time and the longitudinal outcomes, including VA, corneal edema, D-folds, and inflammation. Linear regression models were implemented to analyze the association between operative time and IOP. An ordinary logistic regression model was fit to analyze the association between operative time and patient-reported discomfort. All the models adjusted for patient age, pre-operative logMAR best corrected visual acuity, CDE, nuclear sclerosis grade, pupil dilation, epinephrine, use of iris expansion device, corneal wound suture, intra-operative complication, IFIS, concomitant vision limiting diagnosis, and individual attending. The models were also adjusted for the months after July 1st to control for any inexperience of the resident surgeon in the early months of the academic year. Additionally, the linear mixed effect models were controlled for random intercept and random time effect within patients. A linear regression model was fitted for operative time adjusting attending, Nuclear Sclerosis Grade, Dilation, and IFIS to analyze the association between attending and operative time. Wald test was used to examine the overall difference in operation time between attendings. We also conducted sensitivity analyses by removing attendings with less than 10 cases and the cases with more than 100 min.

Coefficient estimates of operative time and corresponding 95% confidence intervals (CI) and *p*-values were reported. The coefficient estimates can be interpreted as estimated difference in outcome values between patients differing in operative time by 10 min. A positive estimate means that the outcome value would be higher for patients having longer operative time. All analyses were performed in R 4.1.2. Two-sided *p*-value less than 0.05 was considered as statistically significant.

## Results

420 eyes were included in the analysis (Table 1). The mean age of the patient at the time of surgery was 70.5 years. Mean pre-operative best corrected visual acuity was logMAR 0.43 (approximately 20/50 Snellen). 79 eyes (19%) had a concomitant vision limiting diagnosis other than cataracts that could affect their post operative visual potential. The most frequent nuclear sclerotic cataract grade was 2+ (n = 202, 48%).

**Table 1:** Descriptive statistics

Patient age, year, mean (IQR)		70.5 (67–74)
Dilation, mm, mean (IQR)		7.21 (7–8)
Pre-op Best Corrected Visual Acuity, LogMAR, Mean (IQR)		0.43 (0.18–0.48)
Concomitant vision limiting diagnosis, n		79 (19%)
Nuclear sclerosis grade, n		
1+		16 (4%)
1–2+		22 (5%)
2+		202 (48%)
2–3+		66 (16%)
3+		94 (23%)
3–4+		6 (1%)
4+		11 (3%)

**Fig. 1:**
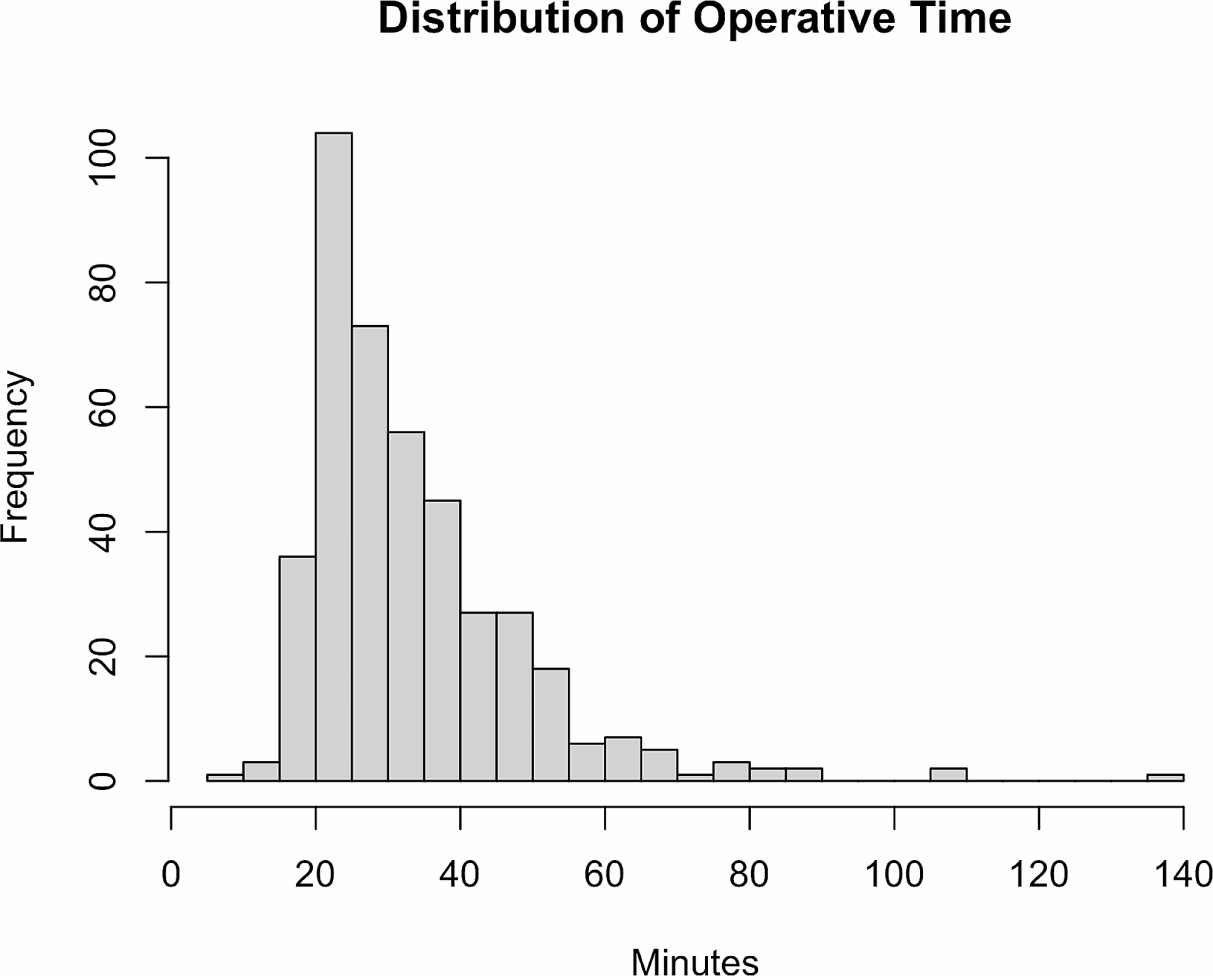
Shows a histogram of resident operative times

Operative factors are described in Table 2. Most surgeries were completed by post-graduate year 4 residents (n = 404, 96%), with a few by post-graduate year 3 residents (n = 15, 4%). The mean months after July 1st was 6.5 months (IQR 3–10). In total, there were 10 different attending physicians that staffed the resident cases, however most cases (n = 368, 88%) were staffed by the three of these attendings. Mean operative time was 34 min (IQR 24–40). Mean CDE was 9.6 (IQR 5.1–12.6). Operative complications including posterior capsular tear, anterior capsular tear, anterior vitrectomy, or zonular dehiscence occurred 11 times (2.6%). Intracameral epinephrine injection was used in 177 cases (42%) while an iris expansion device (Malyugin ring or iris hooks) was used in 27 cases (6%). Wound sutures were placed in 59 cases (14%) in the event of a non-sealing wound or anticipated need for post-operative intravitreal injection. Figure 1 demonstrates the distribution of operative times.

**Table 2:** Operative factors

Operative time, minutes, mean (IQR)	34.1 (24–40)
Cumulative dissipated energy, mean (IQR)	9.6 (5.1–12.6)
Intraoperative complication, n	11 (3%)
Use of iris expansion device, n	27 (6%)
Use of intracameral epinephrine, n	277 (42%)
Wound suture placed, n	
1 suture	53 (13%)
2 sutures	6 (1%)

At POD1, mean IOP was 19 (IQR 15–22) while average pinhole logMAR acuity was 0.30. By use of a multivariable linear regression controlling for attending and operative factors listed above, there was a statistically significant association between operative time and POD1 pinhole visual acuity (*p* = 0.049) (Table 3). The coefficient estimation was 0.0222, indicating longer operative times are associated with higher logMAR POD1 pinhole visual acuity. CDE was significantly associated with operative time (*p* = 0.004), but not with post operative outcomes. At POW1 visual acuity improved to an average of 0.14 logMAR and then to 0.11 logMAR at POM1. Patient discomfort, intraocular pressure, and intraocular inflammation, corneal edema, and Descemet’s membrane folds had no significant association with operative time (Table 3). If we removed the surgeries with more than 100 min operative time, the association of operative time with CDE remained significant (*p* = 0.0008), but not the association with POD1 pinhole visual acuity.

**Table 3:** The effect of operative time on the longitudinal outcomes from linear mixed effects models controlling for attending

Outcome	Estimate (95% confidence interval)			*p*-value	R^2^
POD1 Pinhole LogMAR VA	0.0222	(0.0001,	0.0443)	0.049	0.305
POW1 Best Corrected LogMAR VA	0.0121	(-0.0056,	0.0298)	0.180	0.364
POM1 Best Corrected LogMAR VA	0.0035	(-0.0100,	0.0170)	0.609	0.360
Corneal Edema	0.0317	(-0.0085,	0.0718)	0.128	0.853
D-folds	0.0258	(-0.0007,	0.0523)	0.061	0.679
Intraocular Inflammation	-0.0185	(-0.0514,	0.0145)	0.279	0.637
POD1 discomfort	0.0438	(-0.1985,	0.2860)	0.723	0.136
IOP	-0.0771	(-0.5868,	0.4325)	0.766	0.074

**Fig. 2:**
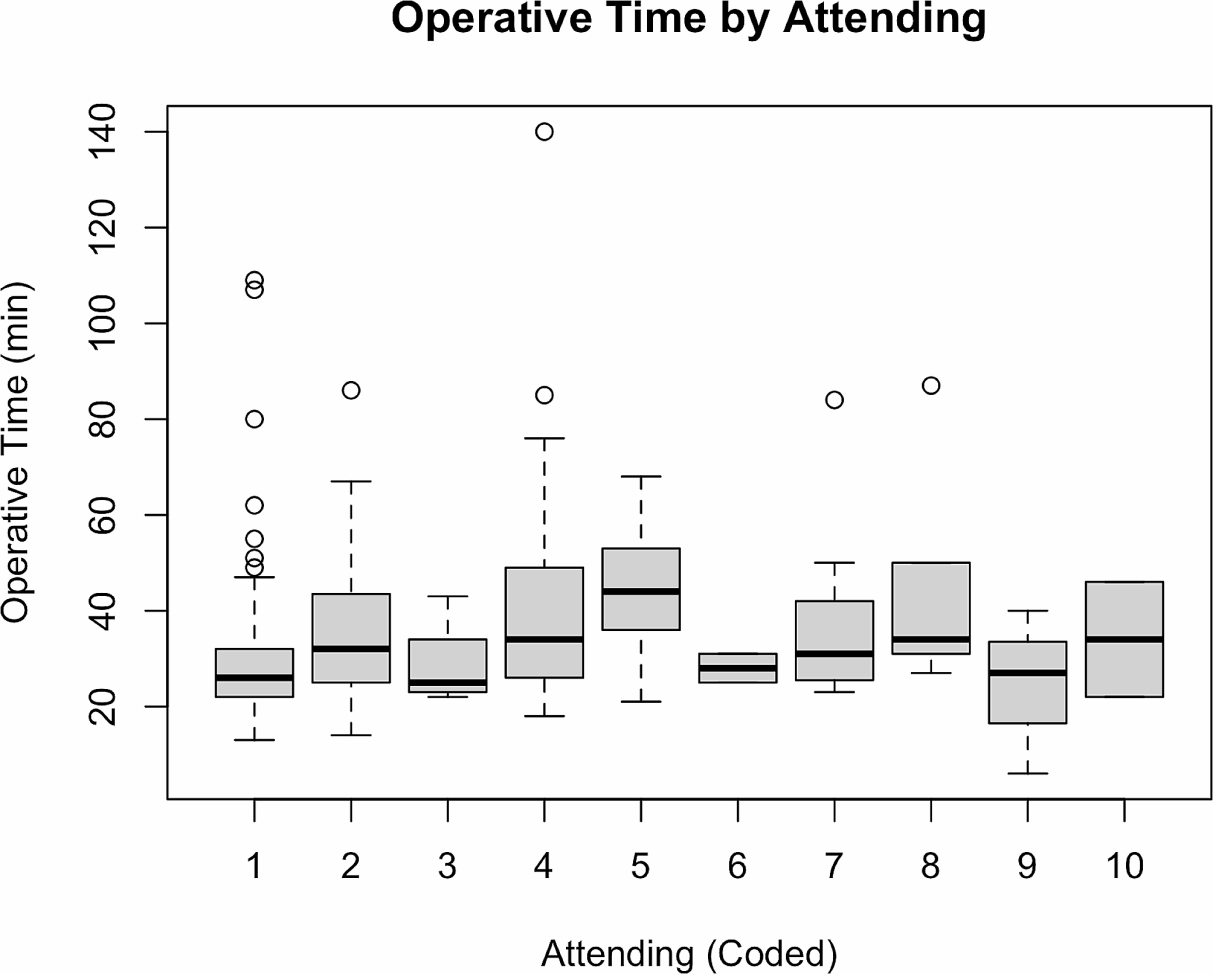
Shows a boxplot of operative times grouped by supervising attending

Figure 2 demonstrates boxplots of the resident operative times grouped by all supervising attending surgeons. Overall operative times varied significantly across supervising attending surgeons (*p* < 0.001), when controlling for complexity of the case, including nuclear sclerosis grade, dilation, and IFIS. The difference remained significant after removing attendings with less than 10 cases (*p* < 0.001, Table 4). Two attendings had statistically shorter mean operative times (29.29 min, *p* < 0.001; 34.82, *p* = 0.013) compared to the attending with the longest operative time (44.67 min) (Table 4). As there were multiple residents who operated with these attendings over the two-year period (five per year), we can presume this effect is independent of the residents’ competency.

**Table 4:** Resident operative time by attending

Attending	Years of Experience	Cases	Min	Max	Median	Mean	Standard deviation
1	26	178	13	109	26	29.29	12.90
2	2	67	14	86	32	34.82	12.34
4	28	123	18	140	34	38.86	8.24
5	16	17	21	68	44	44.64	17.58
7	1	12	23	84	31	37.08	17.19

*P-value for overall attending effect < 0.001 after controlling for case complexity including nuclear sclerosis grade, dilation, and IFIS. *Attending surgeons with < 10 cases were excluded)

## Discussion

In an academic environment, specifically at the Veterans Health Administration, achieving excellent early post operative visual acuity can be a challenge. This is usually thought to be related to increased CDE for junior surgeons who have not learned to maximize phacoemulsification efficiency [8]. Many studies have highlighted phacoemulsification time or CDE as a risk factor for post operative corneal edema and decreased visual acuity [7, 8, 11, 12]. However, few publications have been dedicated to elucidating time as an independent risk factor for poor early post operative visual acuity.

To our knowledge, our study is the first to suggest that operative time is associated with reduced early post-operative visual outcomes, independent of phacoemulsification energy and other key operative factors, specifically in resident cataract surgery. A recent publication by Kalhorn et al. in April 2022 also studied the effect of total operative time on postoperative outcomes. Like our results and that by Reddy et al., Kalhorn showed no association between long surgeries and postoperative inflammation (OR 1.10, *p* = 0.72) [13]. They did report an association between operative time and decreased POD1 visual acuity. However, the authors disclosed they were unable to control for CDE in their multivariate analysis as there were two types of phacoemulsification machines used during the study period. Therefore, their results cannot adjust for the known association between CDE and post operative endothelial loss. Furthermore, they studied cataracts by an attending or fellow surgeon, not a resident. Schmidt et al. also showed an association with worse long term visual outcomes and operative time; however, these results were representative of only patients that required unplanned reoperation by resident surgeons, and not routine cataract surgery [14]. Again, there is no indication that phacoemulsification energy was controlled for. This study also did not explore the attending surgeons’ impact on operative time.

The attending surgeon has a challenging assignment of balancing resident autonomy and patient satisfaction. They have a duty to the patient to safely improve their vision and quality of life, but also to the medical community to effectively train surgical residents to care for the future generation of patients. Studies have shown the safety of cataract surgery by resident physicians is comparable to that of an attending physician [1]. As there was no association with operative time and POW1 or POM1 visual acuity, our data suggests that longer operative time may have no lasting impact on patient outcomes. This supports continued resident autonomy in the operating room.

Our study also suggests that the attending surgeon may be able to reduce resident operative time as several attending surgeons were significantly associated with shorter resident operative time. Our study could not assess the effect of this attending-dependent operative time on resident learning. We would suggest maximizing operating room efficiency to counteract the slower surgical techniques of the surgeon in training. To hasten the procedure, the attending surgeon could assist with tasks that have less educational benefit, including the injection of lidocaine and viscoelastic, hydrating wounds, and checking for the integrity of operative instruments before they are needed. Also, to shorten case time, attendings should feel empowered step in or assist in parts of the procedure where the resident has stopped making adequate progress. It is interestingly worth noting, the mean resident operative time for cataract surgery reported in this study (34.1 min) is significantly shorter than times reported in resident cataract surgery literature (42.3-59.23 min) [14, 15].

By knowing that operative time is associated with decreased early visual acuity, we can effectively counsel the patient on expectations. If the case completed by a more junior resident was particularly longer than average, we may counsel the patient to not expect immediate visual recovery, and therefore mitigate heightened expectations and improve overall satisfaction.

Besides improving early post operative visual recovery, efficiency in the operating room has other beneficial effects. Specifically, efficiency can optimize costs for our healthcare system. A 2014 study estimated the cost due to increased operative time in cases performed by a resident cataract surgeon to be $138,926 over a resident’s 3 years of training [4]. In resource-limited settings such as the VA, shorter operative times can increase case volume and lead to more timely access to care for veterans. Improving cataract surgery efficiency can also improve the resident training experience. An efficient resident and attending team can perform more cataract surgeries per day and increase the resident’s caseload, thereby improving his or her surgical competence after graduation. Studies have shown that quantity of cases is important in the resident learning curve; also signified by the fact that the ACGME has set a hard minimum in case volumes [3, 12, 13]. Improving operative time and thus potentially allowing more cases, may be beneficial not only to the patient but also the trainee [16, 17].

Like most retrospective studies, this study has limitations. First, as there were no control variables, and therefore hidden biases could confound the results. Additionally, postoperative documentation, specifically for that of corneal edema, Descemet’s membrane folds, and inflammation, can be very subjective and without standardization in grading, and could therefore confound the results. Finally, although our results show statistical significance for an association between operative time and POD1 PH VA, the clinical significance could be questioned. It would take a near 45-minute increase in surgical time to affect one line of visual acuity. Further randomized, controlled studies are needed to further elucidate a causal relationship between cataract surgery operative time and early postoperative outcomes.

## Data Availability

The datasets generated and/or analyzed during the current study are not publicly available due to Veterans Affairs policy but are available from the corresponding author on reasonable request.

## Abbreviations

CEIOL: cataract extraction and intraocular lens insertion
POD1: post-operative day 1
POW1: post-operative week 1
POM1: post-operative month 1
VA: visual acuity
CDE: cumulative dissipated energy
PH: pinhole
